# Associations of mind–body integrative care with sleep, metabolic profiles, and pregnancy outcomes in women with gestational diabetes mellitus: A prospective cohort study

**DOI:** 10.1097/MD.0000000000048212

**Published:** 2026-04-17

**Authors:** Ni Xiaowei, Cheng Junli, Ma Shanshan

**Affiliations:** a Department of Gynecology, Shijiazhuang Maternal and Child Health Care Hospital, Shijiazhuang, Hebei Province, China.

**Keywords:** adverse pregnancy outcomes, anxiety, depression, gestational diabetes mellitus, HbA1c, maternal health, mediation analysis, mind–body intervention, psychoneuroimmunology, sleep quality

## Abstract

Sleep and emotional disturbances are common among women with gestational diabetes mellitus (GDM) and are associated with poor glycemic control and adverse pregnancy outcomes (APOs). Mind–body approaches have been proposed as supportive strategies to improve psychological and metabolic health; however, longitudinal evidence during pregnancy remains limited. In this prospective controlled cohort study, 200 women with GDM were enrolled at 24 to 28 gestational weeks. Participants were classified into a mind–body integrative care group (routine prenatal care supplemented with structured mindfulness, relaxation, and sleep management components) or a routine-care group (routine prenatal care alone), according to the care pathway received under routine clinical practice. Outcomes were assessed at baseline, after 8 weeks, and at delivery. Sleep quality (Pittsburgh Sleep Quality Index), anxiety (7-item Generalized Anxiety Disorder Scale), depression (9-item Patient Health Questionnaire), and metabolic indicators (glycated hemoglobin [HbA1c], homeostasis model assessment of insulin resistance, high-sensitivity C-reactive protein) were measured. Multivariable logistic regression, Cox proportional hazards models, and counterfactual-based parallel mediation analyses were applied. After 8 weeks, exposure to mind–body integrative care was associated with lower odds of sleep disturbance (odds ratio = 0.61, 95% confidence interval = 0.38–0.98, *P* = .041) and more favorable metabolic profiles, including lower HbA1c, homeostasis model assessment of insulin resistance, and high-sensitivity C-reactive protein levels. The overall incidence of APOs was lower in the mind–body integrative care group than in the routine-care group (28.0% vs 42.0%, *P* = .038), corresponding to a directionally consistent reduction in APO risk in Cox analysis (hazard ratio ≈ 0.70). Mediation analyses suggested that changes in sleep quality (ΔPittsburgh Sleep Quality Index) and glycemic control (ΔHbA1c) represented the primary indirect pathways, jointly accounting for approximately 67% of the observed association. Exposure to mind–body integrative care was associated with better sleep quality, more favorable metabolic profiles, and a lower incidence of APOs among women with GDM. The observed psychological–metabolic mediation pattern supports the potential relevance of integrative, nonpharmacological approaches in prenatal care while underscoring the need for further randomized studies to establish causality.

## 1. Introduction

Gestational diabetes mellitus (GDM) is a common metabolic disorder in pregnancy, with a global prevalence of 10% to 15% and exceeding 20% in some regions of China.^[1–3]^ Its incidence continues to rise due to increasing obesity, metabolic syndrome, and advanced maternal age. GDM poses substantial short- and long-term risks for both mother and child, including gestational hypertension, cesarean delivery, postpartum type 2 diabetes, preterm birth, macrosomia, neonatal hypoglycemia, and later metabolic disorders.^[4–8]^ Despite advances in screening and therapy, conventional pharmacological or dietary management often fails to address both metabolic instability and psychological distress.^[9–11]^ Factors such as emotional stress, poor adherence, and lifestyle habits remain major challenges, underscoring the need for integrative strategies that target both physiological and psychological dimensions of care.^[12,13]^

Sleep and emotional disturbances are highly prevalent among women with GDM, with approximately 50% to 70% experiencing poor sleep quality and 40% to 50% presenting symptoms of anxiety or depression.^[14–16]^ These conditions are not merely psychological comorbidities but play an active role in GDM pathophysiology.^[17]^ Sleep deprivation and affective stress can trigger hyperactivation of the hypothalamic–pituitary–adrenal (HPA) axis, leading to elevated cortisol levels, systemic inflammation, and insulin resistance.^[18,19]^ Such neuroendocrine–immune dysregulation further impairs glucose metabolism and placental function, creating a vicious cycle that exacerbates maternal metabolic instability and adverse pregnancy outcomes (APOs).^[20,21]^

Mind–body interventions (MBIs) are integrative approaches centered on psychological regulation and supported by techniques such as breathing exercises, mindfulness meditation, relaxation therapy, music therapy, or light physical activity.^[22–24]^ Their primary goal is to enhance emotional stability, reduce sympathetic overactivity, and promote both sleep quality and metabolic homeostasis.^[22,25]^ International studies have demonstrated that mindfulness training, yoga, and cognitive–behavioral therapy effectively alleviate pregnancy-related anxiety and depression.^[24–27]^ Owing to their safety, low cost, and ease of home-based implementation, MBIs represent a promising adjunctive strategy – particularly valuable for pregnant women, in whom pharmacological interventions are limited or undesirable.^[27]^

Recent studies increasingly adopt multilevel frameworks integrating metabolic, psychological, behavioral, and clinical outcome dimensions to better capture the complexity of GDM.^[28]^ However, systematic longitudinal evidence regarding the efficacy and underlying mechanisms of MBIs during pregnancy remains limited. Therefore, building on this emerging psychophysiological framework, we developed a structured mind–body integrative intervention incorporating mindfulness training, relaxation techniques, and sleep hygiene management, and prospectively evaluated its effects on psychological regulation, sleep quality, metabolic remodeling, and pregnancy outcomes in women with GDM.

## 2. Methods

### 2.1. Study design and overall framework

This study was conducted as a prospective controlled cohort study integrating cross-sectional and mediation analyses to evaluate the associations between exposure to a mind–body integrative care program and clinical outcomes among women with GDM. The study was carried out in the Department of Obstetrics at Shijiazhuang Maternal and Child Health Care Hospital between January 2021 and December 2023. Participants were enrolled during mid-pregnancy and followed prospectively until delivery under routine clinical care conditions.

The study protocol was reviewed and approved by the Ethics Committee of Shijiazhuang Maternal and Child Health Care Hospital. All participants provided written informed consent prior to enrollment, in accordance with the Declaration of Helsinki.

### 2.2. Sample size estimation

The sample size was calculated based on the rule of at least 10 events per variable in logistic regression. Assuming an expected incidence of APOs of approximately 30%, an effect size of 0.35, a significance level of α = 0.05, and a statistical power of 0.80, the minimum required sample was estimated at 180 participants. To ensure adequate power and account for potential loss to follow-up, a total of 200 women were enrolled, with 100 in the intervention group and 100 in the control group.

### 2.3. Participants and eligibility criteria

Inclusion criteria are as follows: women aged 18 to 45 years, singleton pregnancy between 24 and 28 gestational weeks, diagnosis of GDM confirmed by the 75-g oral glucose tolerance test based on standard criteria, ability to understand study procedures and provide written informed consent, willingness to participate in the intervention and complete follow-up assessments, and absence of severe psychiatric or systemic illness that could affect participation.

Exclusion criteria are the following: history of preexisting diabetes (type 1 or type 2) or severe insulin resistance; presence of comorbid pregnancy complications, such as thyroid disease, chronic hypertension, or renal dysfunction; current use of antidepressant, antipsychotic, or sedative medications; multiple pregnancies (e.g., twins or higher-order gestations); and incomplete data or loss to follow-up during the study period.

### 2.4. Group classification and care exposure

Participants were classified according to the type of prenatal care received under routine clinical practice, rather than through randomization. Women who received routine antenatal care supplemented with a structured mind–body integrative care program were categorized as the mind–body care group, whereas those who received routine prenatal care and standard health education alone were categorized as the routine-care group. No experimental randomization or investigator-controlled assignment was implemented in this study.

### 2.5. Mind–body integrative care components

The mind–body integrative care program comprised 4 coordinated modules designed to promote psychological balance, improve sleep quality, and enhance metabolic stability. The psychological regulation module included weekly mindfulness meditation sessions (approximately 30 minutes per session), combined with guided breathing and relaxation exercises aimed at strengthening emotional self-regulation and reducing sympathetic overactivation.

The sleep management module focused on sleep hygiene optimization, incorporating bedtime relaxation music, progressive muscle relaxation techniques, and guidance on light exposure and smartphone use before sleep. The health education module provided comprehensive instruction on dietary management, moderate physical activity, and emotional self-care, emphasizing lifestyle consistency and self-monitoring.

In addition, a follow-up and supervision module was implemented as part of routine-care enhancement. Participants were encouraged to maintain online behavioral logs, and trained nurses conducted biweekly follow-up calls to support adherence, address challenges, and provide individualized feedback.

### 2.6. Adherence and participation

Adherence to the mind–body integrative care components was prospectively monitored using predefined session attendance records and online behavioral logs as part of routine-care delivery. Attendance was tracked for each scheduled session, and submission of behavioral or sleep-related logs was recorded throughout the exposure period. For descriptive purposes, adherence was summarized as the number of sessions attended and the proportion of participants submitting logs during the intervention period. Overall, participants completed an average of 6.2 ± 1.4 of the 8 planned sessions, with 82% attending at least 6 sessions, and approximately 75% submitting behavioral or sleep-related logs for more than half of the exposure period. These metrics were used solely to characterize exposure to mind–body integrative care and were not used to define analytic subgroups or to modify the primary analyses.

### 2.7. Care exposure duration and follow-up

Exposure to the mind–body integrative care program lasted approximately 8 weeks, typically from enrollment at 24 to 28 gestational weeks to 34 to 36 weeks of gestation. All participants were subsequently followed prospectively until delivery, and maternal and neonatal outcomes were systematically collected from medical records for analysis.

### 2.8. Assessment timeline and data quality control

Data were collected at 3 standardized time points: T0 (baseline, before intervention), T1 (after 8 weeks of intervention), and T2 (at delivery), enabling assessment of both short-term psychological and metabolic changes and long-term pregnancy outcomes. To ensure data integrity and reliability, all questionnaires were administered by trained research nurses, and laboratory assays were performed in a certified clinical laboratory using standardized protocols. Double data entry and periodic cross-checking were implemented to minimize transcription errors, and missing or inconsistent data were verified against original records before analysis.

### 2.9. Outcome measures and instruments

Because of the behavioral nature of the mind–body integrative care program, blinding of participants and care providers was not feasible. Subjective outcomes, including sleep quality (Pittsburgh Sleep Quality Index [PSQI]) and psychological symptoms (7-item Generalized Anxiety Disorder Scale [GAD-7] and 9-item Patient Health Questionnaire [PHQ-9]), were assessed using standardized, self-administered questionnaires.

APOs were identified from electronic medical records using predefined clinical criteria by personnel not involved in the delivery of mind–body integrative care. To reduce analytic bias, statistical analyses were performed on de-identified datasets, with group labels masked during primary analyses.

### 2.10. Psychological and sleep indicators

Sleep quality was measured using the PSQI, while symptoms of anxiety and depression were evaluated using the GAD-7 and the PHQ-9, respectively.

### 2.11. Metabolic indicators

Fasting plasma glucose, 2-hour postload glucose, glycated hemoglobin (HbA1c), fasting insulin, and insulin resistance index (homeostasis model assessment of insulin resistance [HOMA-IR]) were measured to assess glycemic control. Lipid profiles included total cholesterol, triglycerides (TG), high-density lipoprotein cholesterol, low-density lipoprotein cholesterol, and the TG/high-density lipoprotein (HDL) ratio. Inflammatory markers such as high-sensitivity C-reactive protein (hs-CRP), interleukin-6 (IL-6), and tumor necrosis factor-α (TNF-α) were determined, along with optional endocrine indicators, including leptin, adiponectin, and 25-hydroxyvitamin D.

### 2.12. Pregnancy outcomes (APOs)

APOs included preterm birth, gestational hypertension or preeclampsia, cesarean delivery, fetal distress, low birth weight or macrosomia, neonatal intensive care unit admission, and Apgar score < 7 at 1 minute. These outcomes were verified from medical records at delivery.

## 3. Statistical analysis

Continuous variables were expressed as mean ± standard deviation for normally distributed data or median (interquartile range) for skewed data, while categorical variables were presented as counts and percentages (n [%]). Group comparisons were performed using the Student *t* test, χ^2^ test, or Mann–Whitney *U* test, as appropriate.

Multivariable logistic regression models were used to examine associations between the mind–body intervention and psychological outcomes, including sleep disturbance (PSQI ≥ 7), anxiety (GAD-7 ≥ 5), and depression (PHQ-9 ≥ 5). A hierarchical modeling strategy was adopted – model 1: intervention plus demographic variables (age, gestational week, body mass index [BMI], education); model 2: model 1 + metabolic and biochemical indicators; model 3: model 2 + lifestyle factors (physical activity, caffeine intake, screen time); and model 4: model 3 + clinical covariates (antenatal visits, insulin therapy).

The time to first APO from enrollment to delivery was analyzed using Cox proportional hazards regression. Results were expressed as hazard ratios (HRs) with 95% confidence intervals (CIs). The Kaplan–Meier survival method and log-rank test were applied to compare cumulative event-free probabilities between groups. Covariates were sequentially added to assess the independent effect of the intervention after adjusting for metabolic and psychological factors. To explore potential mechanisms, a parallel multiple mediation analysis was conducted under a counterfactual framework. Indirect, direct, and total effects were estimated using bootstrapping with 5000 resamples, and 95% bias-corrected CIs were calculated. The proportion of mediation was reported for each pathway.

Parallel mediation analyses were conducted to examine whether changes in sleep quality and metabolic indicators mediated the association between exposure to mind–body integrative care and APOs. Mediator changes (Δ scores) were defined as the difference between baseline (T0) and postintervention assessment at 8 weeks (T1). APOs were assessed subsequently at delivery (T2). Thus, all mediation models explicitly followed a temporal sequence of exposure → mediator change (T0–T1) → outcome occurrence (T2).

Participants with incomplete baseline data or missing outcome information due to loss to follow-up were excluded from the primary analyses. Therefore, all analyses were conducted using a complete-case approach, without imputation of missing values.

All analyses were performed using SPSS version 26.0 and R version 4.3.2, with mediation models implemented through the PROCESS macro (model 4). Statistical significance was set at two-tailed *P* < .05. Data visualization, including Kaplan–Meier survival curves and forest plots, was conducted using GraphPad Prism or R ggplot2 packages.

## 4. Results

### 4.1. Baseline characteristics

Of the women initially screened for eligibility (n = 215), 215 met the inclusion criteria and were enrolled. During follow-up, 10 participants were excluded due to incomplete baseline data, and 5 were lost to follow-up before delivery (Fig. S1, Supplemental Digital Content, https://links.lww.com/MD/R643). A total of 200 women with GDM were enrolled, including 100 in the mind–body integrative care group and 100 in the routine-care group. As summarized in Table 1, the 2 groups were comparable in demographic, psychological, and biochemical characteristics (all *P* > .05).

**Table 1:** Baseline characteristics of participants (n = 200).

Variable	Mind–body integrative care group (n = 100)	Routine-care group (n = 100)	*P* value
Age, yr	30.82 ± 4.47	31.27 ± 4.61	.482
Gestational wk	26.13 ± 1.82	26.34 ± 1.93	.416
BMI, kg/m^2^	27.58 ± 3.43	27.91 ± 3.61	.527
Primiparous, n (%)	54 (54.0%)	52 (52.0%)	.765
Previous GDM, n (%)	11 (11.0%)	13 (13.0%)	.676
Smoking, n (%)	2 (2.0%)	3 (3.0%)	.653
Alcohol use, n (%)	1 (1.0%)	2 (2.0%)	.559
PSQI total score	8.64 ± 2.52	8.92 ± 2.63	.494
GAD-7 score	6.08 ± 3.01	6.34 ± 3.08	.574
PHQ-9 score	6.43 ± 3.22	6.66 ± 3.11	.599
FPG, mmol/L	5.38 ± 0.59	5.52 ± 0.61	.265
2h-PG (75-g OGTT), mmol/L	8.79 ± 1.09	8.93 ± 1.12	.348
HbA1c, %	5.91 ± 0.41	6.03 ± 0.40	.124
FINS, µU/mL	14.76 ± 5.53	15.19 ± 5.60	.601
HOMA-IR	3.54 ± 1.43	3.68 ± 1.49	.458
TC, mmol/L	5.63 ± 0.79	5.71 ± 0.84	.532
TG, mmol/L (median [IQR])	2.46 (1.91, 3.07)	2.58 (2.02, 3.15)	.287
HDL-C, mmol/L	1.46 ± 0.25	1.43 ± 0.26	.447
LDL-C, mmol/L	3.01 ± 0.58	3.06 ± 0.61	.572
TG/HDL ratio (median [IQR])	1.67 (1.26, 2.20)	1.79 (1.32, 2.33)	.304
hs-CRP, mg/L (median [IQR])	2.59 (1.73, 3.75)	2.82 (1.90, 4.06)	.231
IL-6, pg/mL (median [IQR])	2.47 (1.74, 3.48)	2.59 (1.84, 3.63)	.343
TNF-α, pg/mL (median [IQR])	1.78 (1.26, 2.37)	1.86 (1.33, 2.46)	.372
ALT, U/L (median [IQR])	17.84 (13.61, 23.28)	18.77 (14.45, 24.49)	.327
AST, U/L (median [IQR])	20.93 (16.81, 25.54)	21.32 (17.03, 25.91)	.476
Leptin, ng/mL (median [IQR])	17.72 (13.06, 23.74)	18.38 (13.58, 24.79)	.428
Adiponectin, µg/mL (median [IQR])	6.94 (5.14, 8.91)	6.75 (5.02, 8.71)	.411
25(OH)D, ng/mL	22.03 ± 5.92	21.48 ± 6.01	.522

Data are expressed as mean ± SD for normally distributed variables and median (IQR) for skewed distributions. *P* values are calculated by Student *t* test, Mann–Whitney *U* test, or χ^2^ test, as appropriate. No significant baseline differences were detected between groups.

2h-PG = 2-hour postload glucose, 25(OH)D = 25-hydroxyvitamin D, ALT = alanine transaminase, AST = aspartate aminotransferase, BMI = body mass index, FINS = fasting insulin, FPG = fasting plasma glucose, GAD-7 = 7-item Generalized Anxiety Disorder Scale, GDM = gestational diabetes mellitus, HbA1c = glycated hemoglobin, HDL-C = high-density lipoprotein cholesterol, HOMA-IR = homeostasis model assessment of insulin resistance, hs-CRP = high-sensitivity C-reactive protein, IL-6 = interleukin-6, IQR = interquartile range, LDL-C = low-density lipoprotein cholesterol, OGTT = oral glucose tolerance test, PHQ-9 = 9-item Patient Health Questionnaire, PSQI = Pittsburgh Sleep Quality Index, TC = total cholesterol, TG = triglycerides, TNF-α = tumor necrosis factor-α.

At baseline, demographic profiles did not differ significantly between groups. The mean age was 30.82 ± 4.47 years in the mind–body integrative care group and 31.27 ± 4.61 years in the routine-care group (*P* = .482). Gestational weeks (26.13 ± 1.82 vs 26.34 ± 1.93) and BMI (27.58 ± 3.43 vs 27.91 ± 3.61 kg/m^2^) were also comparable. The proportions of primiparous women (54.0% vs 52.0%), previous history of GDM (11.0% vs 13.0%), and lifestyle-related factors were similar between groups.

Psychological and sleep-related indicators were well balanced at baseline, with comparable PSQI scores (8.64 ± 2.52 vs 8.92 ± 2.63, *P* = .494), GAD-7 scores (6.08 ± 3.01 vs 6.34 ± 3.08, *P* = .574), and PHQ-9 scores (6.43 ± 3.22 vs 6.66 ± 3.11, *P* = .599).

Metabolic and inflammatory parameters showed no significant differences between groups, including fasting plasma glucose (5.38 ± 0.59 vs 5.52 ± 0.61 mmol/L), 2-hour postload glucose (8.79 ± 1.09 vs 8.93 ± 1.12 mmol/L), HbA1c (5.91 ± 0.41 vs 6.03 ± 0.40%), fasting insulin (14.76 ± 5.53 vs 15.19 ± 5.60 µU/mL), and HOMA-IR (3.54 ± 1.43 vs 3.68 ± 1.49). TG levels were 2.46 (1.91–3.07) versus 2.58 (2.02–3.15) mmol/L, and hs-CRP levels were 2.59 (1.73–3.75) versus 2.82 (1.90–4.06) mg/L (both *P* > .05).

Other lipid indices (high-density lipoprotein cholesterol, low-density lipoprotein cholesterol, TG/HDL ratio), hepatic enzymes (alanine transaminase, aspartate aminotransferase), inflammatory markers (IL-6, TNF-α), and endocrine biomarkers – including leptin (17.72 [13.06–23.74] vs 18.38 [13.58–24.79] ng/mL), adiponectin (6.94 [5.14–8.91] vs 6.75 [5.02–8.71] µg/mL), and 25-hydroxyvitamin D (22.03 ± 5.92 vs 21.48 ± 6.01 ng/mL) – were likewise comparable between the mind–body integrative care group and the routine-care group.

### 4.2. Cross-sectional analysis of sleep disturbance

Multivariable logistic regression was performed to examine the association between exposure to mind–body integrative care and the risk of sleep disturbance (PSQI ≥ 7) among women with GDM. As shown in Table 2, in the initial adjusted model (model 1), which included age, gestational weeks, BMI, and education level, women in the mind–body integrative care group had significantly lower odds of sleep disturbance compared with those in the routine-care group (odds ratio [OR] = 0.62, 95% CI = 0.40–0.95, *P* = .028).

**Table 2:** Multivariable logistic regression analysis for the association between mind–body intervention and sleep disturbance (PSQI ≥ 7) among women with gestational diabetes mellitus.

Variable	Model 1, OR (95% CI)	*P*	Model 2, OR (95% CI)	*P*	Model 3, OR (95% CI)	*P*	Model 4, OR (95% CI)	*P*
Mind–body intervention (yes vs no)	0.62 (0.40–0.95)	.028	0.66 (0.42–1.03)	.067	0.63 (0.40–0.99)	.046	0.61 (0.38–0.98)	.041
Age, yr	0.98 (0.94–1.03)	.468	0.98 (0.94–1.03)	.482	0.98 (0.94–1.03)	.497	0.98 (0.94–1.03)	.505
Gestational wk	0.98 (0.90–1.08)	.704	0.98 (0.90–1.08)	.693	0.98 (0.90–1.08)	.701	0.99 (0.90–1.09)	.861
BMI, kg/m^2^	1.03 (0.98–1.09)	.227	1.03 (0.98–1.09)	.229	1.02 (0.97–1.08)	.402	1.02 (0.97–1.08)	.420
Education (college+ vs ≤HS)	0.88 (0.55–1.40)	.592	0.90 (0.56–1.45)	.663	0.86 (0.53–1.39)	.537	0.86 (0.52–1.41)	.546
HbA1c (*z*)	–	–	1.28 (1.06–1.55)	.010	1.27 (1.05–1.54)	.014	1.28 (1.06–1.55)	.010
HOMA-IR (*z*)	–	–	1.22 (1.02–1.46)	.030	1.20 (1.01–1.44)	.040	1.22 (1.02–1.46)	.030
TG/HDL (*z*)	–	–	1.18 (1.00–1.40)	.049	1.17 (0.99–1.40)	.061	1.18 (1.00–1.40)	.049
hs-CRP (*z*)	–	–	1.20 (1.03–1.40)	.019	1.19 (1.02–1.39)	.027	1.20 (1.03–1.40)	.019
Physical activity (IPAQ-S, *z*)	–	–	–	–	0.87 (0.75–1.02)	.086	0.86 (0.74–1.01)	.066
Caffeine ≥1 cup/d	–	–	–	–	1.10 (0.70–1.73)	.680	1.09 (0.69–1.72)	.710
Screen time ≥2 h/d	–	–	–	–	1.31 (0.86–1.99)	.210	1.32 (0.86–2.02)	.205
Bedtime phone use (yes)	–	–	–	–	1.39 (0.88–2.20)	.156	1.41 (0.89–2.24)	.142
ANC visits (per visit)	–	–	–	–	–	–	0.93 (0.83–1.05)	.248
Insulin therapy (yes)	–	–	–	–	–	–	1.16 (0.66–2.06)	.606
Center fixed effects	–	–	–	–	–	–	Included	–

Outcomes: sleep disturbance (PSQI ≥ 7), anxiety (GAD-7 ≥ 5), and depression (PHQ-9 ≥ 5).

Model 1: adjusted for age, gestational weeks, BMI, and education level.

Model 2: model 1 plus metabolic indicators, including HbA1c (*z*), HOMA-IR (*z*), TG/HDL (*z*), and hs-CRP (*z*).

Model 3: model 2 plus lifestyle factors (physical activity [IPAQ-S, *z*-score], caffeine intake, screen time, and bedtime phone use).

Model 4: model 3 plus ANC visits and insulin therapy.

Variables marked with “(*z*)” were standardized using *z*-score transformation; ORs represent the effect per 1-standard deviation increase.

Bold values indicate *P* < .05.

ANC = antenatal care, BMI = body mass index, CI = confidence interval, GAD-7 = 7-item Generalized Anxiety Disorder Scale, HbA1c = glycated hemoglobin, HDL = high-density lipoprotein, HOMA-IR = homeostasis model assessment of insulin resistance, HS = high school, hs-CRP = high-sensitivity C-reactive protein, IPAQ-S = International Physical Activity Questionnaire-Short Form, OR = odds ratio, PHQ-9 = 9-item Patient Health Questionnaire, PSQI = Pittsburgh Sleep Quality Index, TG = triglycerides.

After sequential adjustment for metabolic, lifestyle, and clinical covariates, this association remained statistically significant in the fully adjusted model (model 4: OR = 0.61, 95% CI = 0.38–0.98, *P* = .041).

Among biochemical indicators, higher HbA1c, HOMA-IR, TG/HDL ratio, and hs-CRP levels were associated with greater odds of sleep disturbance. Each 1-standard deviation increase in HbA1c and HOMA-IR corresponded to 28% (*P* = .010) and 22% (*P* = .030) higher odds of sleep disturbance, respectively, while hs-CRP showed a similar positive association (OR = 1.20, 95% CI = 1.03–1.40, *P* = .019). Greater physical activity was associated with lower odds of sleep disturbance (OR = 0.86, 95% CI = 0.74–1.01, *P* = .066), whereas caffeine intake, screen time, and bedtime phone use demonstrated nonsignificant positive trends.

### 4.3. Cross-sectional analysis of anxiety and depression

Multivariable logistic regression was used to evaluate the associations between exposure to mind–body integrative care and symptoms of anxiety (GAD-7 ≥ 5) and depression (PHQ-9 ≥ 5) among women with GDM. As shown in Tables S1 and S2, Supplemental Digital Content, https://links.lww.com/MD/R644, women in the mind–body integrative care group tended to have lower odds of both anxiety and depressive symptoms compared with those in the routine-care group; however, these associations did not reach conventional statistical significance after full adjustment.

In the fully adjusted model (model 4), the ORs were 0.67 (95% CI = 0.41–1.09, *P* = .104) for anxiety symptoms and 0.64 (95% CI = 0.39–1.04, *P* = .072) for depressive symptoms, respectively.

### 4.4. Distribution of APOs

A total of 200 women with GDM were followed until delivery. As shown in Table 3, the overall incidence of APOs was lower in the mind–body integrative care group than in the routine-care group (28.0% vs 42.0%, *P* = .038).

**Table 3:** Incidence of APOs between groups.

Outcome	Mind–body integrative care group (n = 100)	Routine-care group (n = 100)	χ^2^/*P* value
Any APO (composite)	28 (28.0%)	42 (42.0%)	.038*
Preterm birth (<37 wk)	8 (8.0%)	14 (14.0%)	.168
Gestational hypertension/preeclampsia	9 (9.0%)	15 (15.0%)	.187
Cesarean delivery	36 (36.0%)	44 (44.0%)	.228
Fetal distress	6 (6.0%)	11 (11.0%)	.205
Low birth weight (<2.5 kg)/macrosomia (>4.0 kg)	9 (9.0%)	15 (15.0%)	.197
NICU admission	10 (10.0%)	17 (17.0%)	.153
Apgar < 7 at 1 min	4 (4.0%)	8 (8.0%)	.229

Data are presented as n (%).

*P* values were calculated using the chi-square test for comparisons with adequate expected cell counts and Fisher exact test for outcomes with low expected cell counts.

APO = adverse pregnancy outcome, NICU = neonatal intensive care unit.

* Indicates statistical significance (*P* < .05).

Specifically, women in the mind–body integrative care group exhibited lower incidences of preterm birth (8.0% vs 14.0%), gestational hypertension or preeclampsia (9.0% vs 15.0%), and fetal distress (6.0% vs 11.0%), although these differences did not all reach statistical significance. The rate of cesarean delivery was 36.0% in the mind–body integrative care group and 44.0% in the routine-care group (*P* = .228).

Neonatal outcomes also tended to be more favorable among women in the mind–body integrative care group, including lower proportions of low birth weight or macrosomia (9.0% vs 15.0%) and neonatal intensive care unit admission (10.0% vs 17.0%). The frequency of an Apgar score <7 at 1 minute was 4.0% in the mind–body integrative care group and 8.0% in the routine-care group (*P* = .229).

### 4.5. Prospective analysis of pregnancy outcomes

During follow-up, women exposed to mind–body integrative care exhibited a lower risk of APOs compared with those receiving routine care. In Cox proportional hazards analysis, exposure to mind–body integrative care was associated with a 34% lower risk of APOs in the minimally adjusted model (model 1: HR = 0.66, 95% CI = 0.45–0.96, *P* = .029). This association remained directionally consistent after full adjustment for demographic, metabolic, psychological, and clinical covariates, although it did not reach conventional statistical significance (model 5: HR = 0.71, 95% CI = 0.50–1.01, *P* = .056; Table 4).

**Table 4:** Cox proportional hazards models for time to first APO.

Variable	Model 1, HR (95% CI)	*P*	Model 2, HR (95% CI)	*P*	Model 3, HR (95% CI)	*P*	Model 4, HR (95% CI)	*P*	Model 5, HR (95% CI)	*P*
Mind–body intervention (yes vs no)	0.66 (0.45–0.96)	.029	0.68 (0.46–1.01)	.056	0.70 (0.49–0.99)	.043	0.72 (0.51–1.02)	.065	0.71 (0.50–1.01)	.056
Age, yr	–	–	1.01 (0.98–1.05)	.474	1.01 (0.98–1.05)	.493	1.01 (0.98–1.05)	.498	1.01 (0.98–1.05)	.510
Gestational wk at enrollment	–	–	0.98 (0.92–1.05)	.556	0.98 (0.92–1.05)	.560	0.98 (0.92–1.05)	.568	0.98 (0.92–1.05)	.572
BMI, kg/m^2^	–	–	1.02 (0.99–1.06)	.162	1.02 (0.99–1.06)	.176	1.02 (0.99–1.06)	.183	1.02 (0.99–1.06)	.186
Primiparous (yes)	–	–	0.91 (0.63–1.33)	.640	0.90 (0.62–1.31)	.586	0.90 (0.62–1.31)	.592	0.91 (0.63–1.32)	.641
HbA1c (*z*)	–	–	–	–	1.22 (1.04–1.43)	.017	1.20 (1.02–1.41)	.025	1.18 (1.01–1.39)	.043
HOMA-IR (*z*)	–	–	–	–	1.18 (1.01–1.38)	.037	1.16 (0.99–1.36)	.067	1.14 (0.97–1.34)	.110
TG/HDL (*z*)	–	–	–	–	1.15 (0.99–1.33)	.064	1.14 (0.98–1.32)	.088	1.13 (0.97–1.31)	.112
hs-CRP (*z*)	–	–	–	–	1.20 (1.03–1.41)	.019	1.19 (1.02–1.39)	.028	1.18 (1.01–1.38)	.040
ΔHbA1c (*z*)	–	–	–	–	–	–	1.17 (1.01–1.36)	.038	1.16 (1.00–1.35)	.048
ΔHOMA-IR (*z*)	–	–	–	–	–	–	1.14 (0.99–1.32)	.069	1.12 (0.97–1.30)	.121
PSQI at baseline (per 1 unit)	–	–	–	–	–	–	–	–	1.04 (1.00–1.09)	.058
GAD-7 at baseline (per 1 unit)	–	–	–	–	–	–	–	–	1.03 (0.99–1.07)	.111
PHQ-9 at baseline (per 1 unit)	–	–	–	–	–	–	–	–	1.02 (0.98–1.06)	.295

Outcome: time to first APO, defined as a composite of preterm birth, gestational hypertension/preeclampsia, cesarean delivery, fetal distress, low birth weight/macrosomia, NICU admission, or Apgar score < 7 at 1 min.

Continuous predictors were standardized using *z*-score transformation; HRs reflect the effect per 1-standard deviation increase.

The proportional hazards assumption was assessed using Schoenfeld residuals and was satisfied (global test *P* = .610).

Bold values indicate *P* < .05.

APO = adverse pregnancy outcome, BMI = body mass index, CI = confidence interval, GAD-7 = 7-item Generalized Anxiety Disorder Scale, HbA1c = glycated hemoglobin, HDL = high-density lipoprotein, HOMA-IR = homeostasis model assessment of insulin resistance, HR = hazard ratio, HS = high school, hs-CRP = high-sensitivity C-reactive protein, PHQ-9 = 9-item Patient Health Questionnaire, PSQI = Pittsburgh Sleep Quality Index, TG = triglycerides.

**Figure 1.**
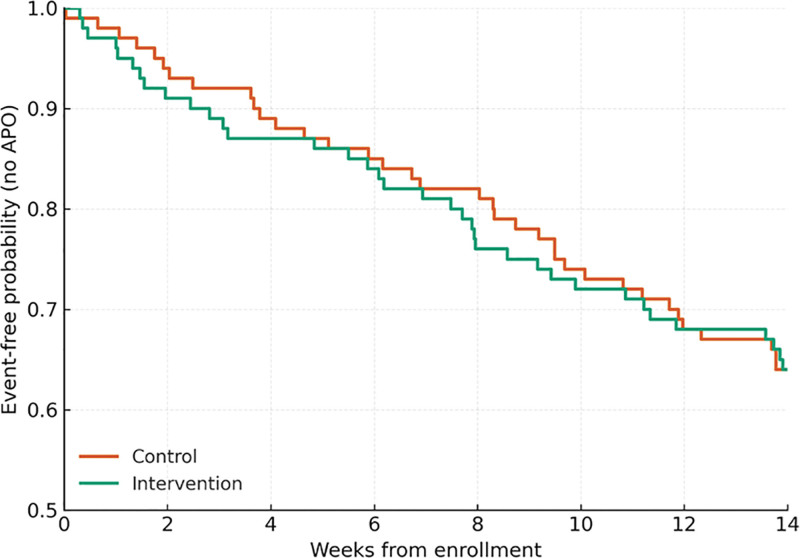
Kaplan–Meier curves of time to first APO by intervention status. Kaplan–Meier survival curves showing event-free probability (no APO) from enrollment to delivery. The intervention group (green) received mind–body management, while the control group (orange) received routine care. Censoring was applied at 14 weeks after enrollment. Differences between groups were evaluated using the log-rank test (two-sided). APO = adverse pregnancy outcome.

As illustrated in Figure 1, women in the mind–body integrative care group maintained a higher event-free probability throughout gestation compared with those in the routine-care group, with survival curves diverging progressively toward delivery. The between-group difference was statistically significant based on the log-rank test (*P* = .031), indicating a lower cumulative incidence of APOs among women receiving mind–body integrative care.

### 4.6. Parallel mediation analysis

Changes in sleep quality and metabolic indicators from baseline (T0) to the postexposure assessment at 8 weeks (T1) were modeled as potential mediators of APOs occurring later in pregnancy (T2). Parallel mediation analyses suggested that changes in psychological and metabolic parameters partially explained the observed association between exposure to mind–body integrative care and a lower risk of APOs.

As shown in Table 5, the total indirect effect across all mediators was statistically significant (*a* × *b* = –0.23, 95% CI = −0.38 to −0.10, *P* < .001), accounting for approximately 67% of the total association. Among individual pathways, changes in sleep quality (ΔPSQI) and glycemic control (ΔHbA1c) represented the largest indirect components, contributing 18% and 21% of the total indirect association, respectively. Changes in insulin resistance (ΔHOMA-IR) and anxiety symptoms (ΔGAD-7) also demonstrated significant indirect associations, whereas changes in depressive symptoms (ΔPHQ-9) showed a weaker, borderline indirect association.

**Table 5:** Parallel mediation analysis of mind–body intervention, changes in psychological/sleep and metabolic indicators, and adverse pregnancy outcomes.

Pathway (mediator)	Indirect effect (*a* × *b*)	95% CI	Proportion mediated (%)	Significance
ΔPSQI (change in sleep quality)	−0.06	−0.12 to −0.02	18.2%	*P* = .012
ΔGAD-7 (change in anxiety)	−0.03	−0.08 to −0.01	9.5%	*P* = .034
ΔPHQ-9 (change in depression)	−0.02	−0.06 to 0.00	6.7%	*P* = .061
ΔHbA1c (change in glycemic control)	−0.07	−0.14 to −0.03	21.3%	*P* = .009
ΔHOMA-IR (change in insulin resistance)	−0.05	−0.11 to −0.02	15.6%	*P* = .018
Total indirect effect (all mediators)	−0.23	−0.38 to −0.10	67.0%	*P* < .001
Direct effect (*c*′)	−0.11	−0.27 to 0.03	–	*P* = .112
Total effect (*c*)	−0.34	−0.54 to −0.16	100%	*P* < .001

Models were adjusted for age, gestational weeks at enrollment, BMI, education level, baseline HbA1c, and baseline psychological and sleep indicators.

Δ denotes the change from baseline (T0) to postintervention assessment at 8 weeks (T1).

Indirect effects were estimated under the counterfactual framework, and 95% CIs were obtained using nonparametric bootstrapping with 5000 resamples.

Bold values indicate statistically significant mediation effects (*P* < .05).

BMI = body mass index, CI = confidence interval, GAD-7 = 7-item Generalized Anxiety Disorder Scale, HbA1c = glycated hemoglobin, HOMA-IR = homeostasis model assessment of insulin resistance, PHQ-9 = 9-item Patient Health Questionnaire, PSQI = Pittsburgh Sleep Quality Index.

After inclusion of all mediators, the direct association between exposure to mind–body integrative care and APOs was attenuated and no longer statistically significant (*c*′ = −0.11, 95% CI = −0.27 to 0.03), consistent with a pattern of partial mediation rather than a fully direct association.

## 5. Discussion

This prospective cohort study evaluated the associations between exposure to mind–body integrative care and sleep quality, psychological status, metabolic parameters, and pregnancy outcomes in women with GDM, and explored potential pathways using parallel mediation analysis. Women exposed to mind–body integrative care exhibited a lower likelihood of sleep disturbance, with an approximately 40% reduction in the odds of poor sleep quality. Symptoms of anxiety and depression showed consistent downward trends, although these associations did not reach conventional statistical significance. Favorable differences were also observed in metabolic indicators, including lower levels of HbA1c, HOMA-IR, and hs-CRP. In addition, exposure to mind–body integrative care was associated with a lower risk of APOs. Mediation analyses further suggested that changes in sleep quality (ΔPSQI) and glycemic control (ΔHbA1c) represented the primary indirect pathways linking exposure to mind–body integrative care with pregnancy outcomes, jointly accounting for approximately 67% of the observed association. Collectively, these findings support a psychological–metabolic coupling pattern in which improvements in sleep and metabolic regulation are statistically associated with more favorable pregnancy outcomes among women with GDM.

Sleep disturbance is highly prevalent among women with GDM and is closely linked to poor glycemic control, inflammation, and APOs.^[29–31]^ Disrupted sleep exacerbates insulin resistance and hormonal imbalance, forming a vicious cycle that worsens metabolic instability.^[32–34]^ Despite this, few interventions specifically target sleep and psychological health in GDM, and most current management focuses solely on diet and medication.^[35–37]^ Therefore, integrating mind–body approaches that address both psychological regulation and sleep quality is essential to achieve comprehensive metabolic and obstetric benefits.

The present study demonstrated that the mind–body intervention significantly improved sleep quality among women with GDM, which is consistent with previous evidence from mindfulness-based and relaxation training programs in pregnant populations.^[38–42]^ The potential mechanisms may involve reduced sympathetic activation and attenuation of HPA axis hyperactivity, leading to lower stress hormone levels, enhanced melatonin secretion, and improved circadian rhythm stability.^[11–13,29,43–47]^ In addition, mindfulness practices may strengthen emotional self-regulation, enabling pregnant women to better manage anxiety-provoking stimuli.^[30,48,49]^ Although anxiety and depression scores showed a decreasing trend without statistical significance, this may be attributed to the relatively short 8-week intervention period, the lagging nature of emotional improvement compared with physiological changes, and the limited sample size that constrained statistical power.

Women in the mind–body integrative care group exhibited a lower incidence of APOs, particularly preterm birth, fetal distress, and hypertensive disorders of pregnancy, compared with those in the routine-care group. The consistent results observed in both the Cox proportional hazards model and Kaplan–Meier survival analysis strengthen the reliability of these findings. These protective effects may be explained by multiple interconnected mechanisms. By improving metabolic control and reducing systemic inflammation, the intervention likely alleviated placental oxidative stress and enhanced uteroplacental function.^[25,50]^ At the same time, stabilization of maternal glucose levels and emotional fluctuations contributed to a more favorable intrauterine environment.^[51]^ In addition, the observed reductions in HbA1c and HOMA-IR indicate that the mind–body intervention not only improved psychological well-being but also promoted glycemic and metabolic stability. Improved sleep quality can enhance insulin sensitivity and glucose regulation, while reduced anxiety and stress may suppress activation of inflammatory pathways involving IL-6, TNF-α, and CRP.^[15,32]^ Furthermore, the integrative program encouraged healthier lifestyle behaviors, including balanced nutrition, moderate physical activity, and greater self-management awareness, which together contributed to better metabolic outcomes in women with GDM.^[33–35]^

Notably, the association between exposure to mind–body integrative care and APOs was attenuated after further adjustment for sleep quality and psychological variables in the fully adjusted Cox model, resulting in a marginally nonsignificant HR. Rather than weakening the overall interpretation, this attenuation is consistent with the proposed mediation hypothesis, suggesting that improvements in sleep and psychological regulation may account for a substantial proportion of the observed association. This pattern aligns with the mediation analyses, which demonstrated significant indirect effects through changes in sleep quality and glycemic control, supporting a psychophysiological pathway linking mind–body care exposure to pregnancy outcomes.

The mediation analysis indicated that changes in sleep quality (ΔPSQI) and glycemic control (ΔHbA1c) represented the most prominent indirect pathways, accounting for approximately 18% and 21% of the total association, respectively. Additional, albeit smaller, indirect contributions were observed for changes in insulin resistance (ΔHOMA-IR) and anxiety symptoms (ΔGAD-7), whereas changes in depressive symptoms (ΔPHQ-9) demonstrated only a borderline association. Collectively, the indirect effects explained approximately 67% of the total association between exposure to mind–body integrative care and APOs, suggesting that a substantial proportion of this relationship may operate through psychological–metabolic coupling.^[44,52–54]^

From a mechanistic perspective, these findings are consistent with a conceptual cascade in which improvements in psychological regulation and sleep quality are linked to downstream metabolic adaptations. Prior evidence suggests that enhanced sleep and stress regulation may be associated with improved HPA axis feedback, reduced inflammatory activity, and greater insulin sensitivity, which together may contribute to more favorable pregnancy outcomes.^[55–57]^. Importantly, these pathways should be interpreted as biologically plausible associations rather than confirmed causal mechanisms. Taken together, the present findings support an integrative psychoneuroimmunological framework in which psychological, sleep-related, and metabolic processes are interrelated during pregnancy.^[26,51,58]^ While this study provides longitudinal evidence consistent with a dual psychological–metabolic mediation pattern, further randomized and mechanistic studies are required to confirm causality and to refine this multidimensional biopsychological model for maternal health management.

The findings of this study underscore the potential clinical value of mind–body integrative care as a safe, low-cost, and feasible nonpharmacological strategy for women with GDM. Rather than serving as a stand-alone treatment, such approaches may function as a supportive adjunct to routine prenatal care, with particular relevance for addressing sleep-related and psychosocial dimensions alongside metabolic regulation. Importantly, the observed associations were primarily driven by improvements in sleep quality, which appeared to play a central role in the proposed psychophysiological pathway. Given the constraints and cautious use of pharmacological therapies during pregnancy, brief, structured mind–body practices – such as mindfulness, relaxation techniques, and sleep hygiene guidance – may be pragmatically integrated into prenatal visits or delivered through home-based or digital platforms. Nevertheless, in light of the nonrandomized design, these findings should be interpreted as associative, and further randomized trials are required to establish causal effects and define optimal implementation strategies.

This study has several important limitations that should be acknowledged. First, although the study was conducted prospectively, it was a single-center investigation with a relatively modest sample size, which may limit generalizability. More importantly, group classification was based on routine clinical care pathways rather than randomization, and therefore residual selection bias and unmeasured confounding – such as differences in health literacy, social support, or motivation – cannot be fully excluded, despite comparable baseline characteristics and multivariable adjustment. In addition, participant blinding was not feasible given the behavioral nature of the intervention, which may increase the risk of performance and detection bias, particularly for self-reported outcomes such as sleep quality and psychological symptoms. Although APOs were ascertained from medical records and statistical analyses were conducted using de-identified data, these measures cannot entirely eliminate bias. Second, the intervention duration was relatively short, and improvements in emotional outcomes such as anxiety and depressive symptoms may require longer exposure or sustained reinforcement. Third, while the composite APO was prespecified as the primary endpoint, analyses of individual outcome components involved multiple comparisons and should therefore be interpreted cautiously, as no formal correction for multiple testing was applied. Fourth, the study relied primarily on self-reported instruments (e.g., PSQI, GAD-7, PHQ-9) to assess key psychological and sleep-related mediators. Although these tools are well validated, reporting bias cannot be excluded, and the absence of objective sleep or stress-related physiological measures limits mechanistic resolution. In addition, the study did not include detailed assessments of hormonal or inflammatory mediators (e.g., cortisol or cytokine profiles), which could further elucidate the biological pathways linking MBIs to metabolic and obstetric outcomes. Partial loss to follow-up may also have contributed to the underestimation of long-term effects.

Future research should prioritize multicenter randomized controlled trials with adequate power, longer intervention durations, and rigorous outcome assessment to establish causality and validate these findings. Extended follow-up into the postpartum period, incorporation of objective monitoring technologies (such as wearable devices and mobile health applications), and integration of multi-omic and neurobiological markers may further clarify mechanisms and inform scalable, personalized implementation strategies.

## 6. Conclusion

This study demonstrates that exposure to a structured mind–body integrative care program, incorporating modules targeting mindfulness, relaxation, and sleep management, was associated with improved sleep quality, favorable psychological profiles, enhanced metabolic stability, and a lower risk of APOs among women with GDM. The observed psychological–metabolic dual mediation pattern highlights sleep-centered psychophysiological regulation as a key pathway linking mind–body care exposure to pregnancy outcomes, providing supportive evidence for integrated, nonpharmacological prenatal management. These findings lay the groundwork for future randomized controlled trials and the development of personalized, scalable mind–body intervention strategies in prenatal care.

## Acknowledgments

The authors sincerely thank all study participants for their invaluable contributions.

## Author contributions

**Conceptualization:** Ni Xiaowei, Cheng Junli, Ma Shanshan.

## Supplementary Material




