# Supplementary figures and images for: Associations of mind–body integrative care with sleep, metabolic profiles, and pregnancy outcomes in women with gestational diabetes mellitus: A prospective cohort study
Source: Medicine (Baltimore). 2026 Apr 17;105(16):e48212. doi: 10.1097/MD.0000000000048212 (PMC13095326; doi:10.1097/MD.0000000000048212)

**Supplementary Figure 1. Participant flow diagram of the prospective cohort study**

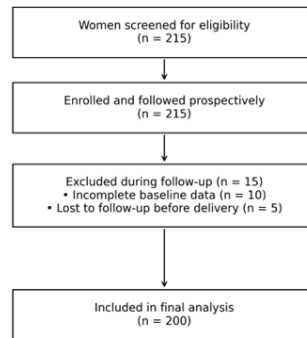

Supplement: Supplementary file 1 [file medi-105-e48212-s001.pdf]
