# Supplementary material for: Associations of mind–body integrative care with sleep, metabolic profiles, and pregnancy outcomes in women with gestational diabetes mellitus: A prospective cohort study
Source: Medicine (Baltimore). 2026 Apr 17;105(16):e48212. doi: 10.1097/MD.0000000000048212 (PMC13095326; doi:10.1097/MD.0000000000048212)
Supplement: Supplementary file 2 [file medi-105-e48212-s002.pdf]

Supplementary Table S1. Multivariable logistic regression for anxiety symptoms (GAD-7 ≥ 5) in women with gestational diabetes mellitus.

**Variable**	**Model 1 OR (95% CI)**	***P***	**Model 2 OR (95% CI)**	***P***	**Model 3 OR (95% CI)**	***P***	**Model 4 OR (95% CI)**	***P***
Mind–body intervention	0.70 (0.45–1.08)	0.106	0.73 (0.46–1.16)	0.183	0.69 (0.43–1.11)	0.125	0.67 (0.41–1.09)	0.104
Age, years	0.99 (0.95–1.03)	0.595	0.99 (0.95–1.03)	0.622	0.99 (0.95–1.04)	0.720	0.99 (0.95–1.04)	0.733
Gestational weeks	0.99 (0.91–1.08)	0.785	0.99 (0.91–1.08)	0.802	0.99 (0.91–1.08)	0.807	1.00 (0.92–1.09)	0.980
BMI, kg/m²	1.02 (0.97–1.07)	0.452	1.02 (0.97–1.07)	0.470	1.01 (0.96–1.07)	0.680	1.01 (0.96–1.07)	0.685
Education (College+ vs ≤HS)	0.91 (0.58–1.43)	0.679	0.93 (0.59–1.47)	0.755	0.88 (0.55–1.42)	0.601	0.88 (0.54–1.42)	0.598
**HbA1c (z)**	—	—	**1.24 (1.04–1.49)**	**0.019**	**1.23 (1.03–1.48)**	**0.024**	**1.24 (1.04–1.49)**	**0.019**
**HOMA-IR (z)**	—	—	1.17 (0.98–1.40)	0.082	1.16 (0.97–1.39)	0.107	1.17 (0.98–1.40)	0.082
**TG/HDL (z)**	—	—	**1.19 (1.01–1.41)**	**0.036**	**1.18 (1.00–1.40)**	**0.049**	**1.19 (1.01–1.41)**	**0.036**
**hs-CRP (z)**	—	—	**1.21 (1.04–**	**0.014**	**1.20 (1.03–**	**0.018**	**1.21 (1.04–1.41)**	**0.014**

**Variable**	**Model 1 OR (95% CI)**	***P***	**Model 2 OR (95% CI)**	***P***	**Model 3 OR (95% CI)**	***P***	**Model 4 OR (95% CI)**	***P***
			**1.41)**		**1.40)**			
Physical activity (IPAQ-S, z)	—	—	—	—	**0.84 (0.72–0.98)**	**0.028**	**0.84 (0.72–0.98)**	**0.028**
Caffeine ≥1 cup/day	—	—	—	—	1.06 (0.69–1.64)	0.790	1.05 (0.67–1.63)	0.822
Screen time ≥2 h/day	—	—	—	—	1.33 (0.88–2.02)	0.175	1.35 (0.89–2.06)	0.158
Bedtime phone use (Yes)	—	—	—	—	1.43 (0.91–2.24)	0.121	1.44 (0.92–2.27)	0.111
ANC visits (per visit)	—	—	—	—	—	—	0.95 (0.86–1.06)	0.339
Insulin therapy (Yes)	—	—	—	—	—	—	1.10 (0.60–2.02)	0.747
Center fixed effects	—	—	—	—	—	—	Included	—

Notes:

- Outcomes: sleep disturbance (PSQI ≥ 7), anxiety (GAD-7 ≥ 5), depression (PHQ-9 ≥ 5).
- Model 1: intervention + age, gestational weeks, BMI, education.
- Model 2: Model 1 + **HbA1c (z)**, **HOMA-IR (z)**, **TG/HDL (z)**, **hs-CRP (z)** (all standardized; ORs per +1 SD).
- Model 3: Model 2 + lifestyle (IPAQ-S z-score, caffeine, screen time, bedtime phone

use).

- Model 4: Model 3 + ANC visits, insulin therapy, and center/batch fixed effects (if multicenter).
- Bold indicates $P < 0.05$. OR and 95% CI rounded to two decimals; P to three decimals.

Supplementary Table S2. Multivariable logistic regression for depressive symptoms (PHQ-9 ≥ 5) in women with gestational diabetes mellitus.

**Variable**	**Model 1 OR (95% CI)**	***P***	**Model 2 OR (95% CI)**	***P***	**Model 3 OR (95% CI)**	***P***	**Model 4 OR (95% CI)**	***P***
Mind–body intervention	0.68 (0.44–1.06)	0.088	0.70 (0.45–1.11)	0.126	0.66 (0.41–1.05)	0.079	0.64 (0.39–1.04)	0.072
Age, years	0.99 (0.95–1.03)	0.569	0.99 (0.95–1.03)	0.598	0.99 (0.95–1.04)	0.733	0.99 (0.95–1.04)	0.742
Gestational weeks	1.00 (0.92–1.09)	0.978	1.00 (0.92–1.09)	0.982	1.00 (0.92–1.09)	0.990	1.01 (0.93–1.10)	0.834
BMI, kg/m²	1.02 (0.97–1.08)	0.431	1.02 (0.97–1.08)	0.443	1.01 (0.96–1.07)	0.719	1.01 (0.96–1.07)	0.724
Education (College+ vs ≤HS)	0.90 (0.57–1.44)	0.674	0.92 (0.58–1.48)	0.740	0.88 (0.54–1.43)	0.611	0.88 (0.54–1.44)	0.628
**HbA1c (z)**	—	—	**1.20 (1.00–1.44)**	**0.050**	**1.19 (0.99–1.43)**	0.062	**1.20 (1.00–1.44)**	**0.050**
**HOMA-IR (z)**	—	—	1.19 (0.99–1.43)	0.062	1.18 (0.98–1.42)	0.086	1.19 (0.99–1.43)	0.062
**TG/HDL (z)**	—	—	1.16 (0.98–1.38)	0.085	1.15 (0.97–1.37)	0.106	1.16 (0.98–1.38)	0.085
**hs-CRP (z)**	—	—	**1.23 (1.06–**	**0.008**	**1.22 (1.05–**	**0.011**	**1.23 (1.06–1.44)**	**0.008**

**Variable**	**Model 1 OR (95% CI)**	***P***	**Model 2 OR (95% CI)**	***P***	**Model 3 OR (95% CI)**	***P***	**Model 4 OR (95% CI)**	***P***
			**1.44)**		**1.42)**			
Physical activity (IPAQ-S, z)	—	—	—	—	**0.83 (0.71–0.97)**	**0.019**	**0.82 (0.70–0.96)**	**0.014**
Caffeine ≥1 cup/day	—	—	—	—	1.07 (0.70–1.63)	0.752	1.08 (0.70–1.66)	0.742
Screen time ≥2 h/day	—	—	—	—	1.36 (0.90–2.06)	0.140	1.38 (0.90–2.10)	0.136
Bedtime phone use (Yes)	—	—	—	—	1.46 (0.94–2.29)	0.093	1.47 (0.94–2.31)	0.092
ANC visits (per visit)	—	—	—	—	—	—	0.94 (0.85–1.04)	0.286
Insulin therapy (Yes)	—	—	—	—	—	—	1.12 (0.63–2.00)	0.708
Center fixed effects	—	—	—	—	—	—	Included	—

Notes:

- Outcomes: sleep disturbance (PSQI ≥ 7), anxiety (GAD-7 ≥ 5), depression (PHQ-9 ≥ 5).
- Model 1: intervention + age, gestational weeks, BMI, education.
- Model 2: Model 1 + **HbA1c (z)**, **HOMA-IR (z)**, **TG/HDL (z)**, **hs-CRP (z)** (all standardized; ORs per +1 SD).
- Model 3: Model 2 + lifestyle (IPAQ-S z-score, caffeine, screen time, bedtime phone use).

- Model 4: Model 3 + ANC visits, insulin therapy, and center/batch fixed effects (if multicenter).
- Bold indicates $P < 0.05$. OR and 95% CI rounded to two decimals; P to three decimals.
